# 3D Multi-Cell Simulation of Tumor Growth and Angiogenesis

**DOI:** 10.1371/journal.pone.0007190

**Published:** 2009-10-16

**Authors:** Abbas Shirinifard, J. Scott Gens, Benjamin L. Zaitlen, Nikodem J. Popławski, Maciej Swat, James A. Glazier

**Affiliations:** The Biocomplexity Institute and Department of Physics, Indiana University Bloomington, Bloomington, Indiana, United States of America; University of Birmingham, United Kingdom

## Abstract

We present a 3D multi-cell simulation of a generic simplification of vascular tumor growth which can be easily extended and adapted to describe more specific vascular tumor types and host tissues. Initially, tumor cells proliferate as they take up the oxygen which the pre-existing vasculature supplies. The tumor grows exponentially. When the oxygen level drops below a threshold, the tumor cells become hypoxic and start secreting pro-angiogenic factors. At this stage, the tumor reaches a maximum diameter characteristic of an avascular tumor spheroid. The endothelial cells in the pre-existing vasculature respond to the pro-angiogenic factors both by chemotaxing towards higher concentrations of pro-angiogenic factors and by forming new blood vessels via angiogenesis. The tumor-induced vasculature increases the growth rate of the resulting vascularized solid tumor compared to an avascular tumor, allowing the tumor to grow beyond the spheroid in these linear-growth phases. First, in the linear-spherical phase of growth, the tumor remains spherical while its volume increases. Second, in the linear-cylindrical phase of growth the tumor elongates into a cylinder. Finally, in the linear-sheet phase of growth, tumor growth accelerates as the tumor changes from cylindrical to paddle-shaped. Substantial periods during which the tumor grows slowly or not at all separate the exponential from the linear-spherical and the linear-spherical from the linear-cylindrical growth phases. In contrast to other simulations in which avascular tumors remain spherical, our simulated avascular tumors form cylinders following the blood vessels, leading to a different distribution of hypoxic cells within the tumor. Our simulations cover time periods which are long enough to produce a range of biologically reasonable complex morphologies, allowing us to study how tumor-induced angiogenesis affects the growth rate, size and morphology of simulated tumors.

## Introduction

### Biology Background

The development of a primary solid tumor starts from a single cell that proliferates in an inappropriate manner, dividing repeatedly to form a cluster of tumor cells. Nutrient and waste diffusion lengths limit the size of such *avascular tumor spheroids* to about 1 mm [1]. The central region of the spheroid becomes necrotic, with a surrounding layer of cells whose hypoxia triggers a cascade of hypoxia-inducible factor-1 (*HIF-1*) and vascular-endothelial-growth-factor (*VEGF*)-mediated signaling events that initiate tumor vascularization by promoting growth and extension (*angiogenesis*) of nearby blood vessels [2]. This general sequence occurs in many types of tumors. Vascularized tumors are able to grow to a much larger size than spheroids and are more likely to spread and metastasize using blood vessels as pathways for invasion [3].

Both fetal and adult angiogenesis is primarily a response to hypoxia [2], [4]–[7]. In adults, angiogenesis plays key roles during tissue repair and remodeling, *e.g.* during wound healing and expansion of tissues during the female reproductive cycle.

The level of HIF-1*α* DNA-binding activity in the nucleus varies exponentially with oxygen tension over the physiological range in mammalian cells [8]. Cells exposed to hypoxic conditions accumulate activated HIF-1*α* in their nuclei in less than 2 minutes [9]. HIF-1*α* changes the expression levels of numerous hypoxia-dependent genes including those responsible for oxygen transport, vascular regulation, angiogenesis, glucose uptake, and glycolysis (reviewed in [10]). HIF activation also upregulates key angiogenesis regulatory signaling molecules including VEGF-A and its receptors VEGFR-1 and VEGFR-2 [2]. VEGF-A has diffusive, ECM-bound, and semi-ECM-bound isoforms which differ in weight and heparin-binding affinity [11]. Tumors secreting different VEGF-A isoforms induce the formation of morphologically different neo-vascular beds [12].

Endothelial cells form two distinct types which respond differently to VEGF-A. Non-dividing *tip cells* form filopodia and migrate towards sources of VEGF-A, while non-migrating *stalk cells* proliferate but do not form filopodia. [13]–[15]. Two cell types are functionally necessary because if every endothelial cell were a tip cell, the vessels would disintegrate, while uniform division of stalk cells would fail to form vessels in the correct pattern [13], [14].

Cell-adhesion plays a crucial role in the formation and stabilization of nascent blood vessels (see [16], [17] for reviews). Formation of cell-cell adhesion junctions via cadherins like VE-cadherin inhibits the chemotaxis response of endothelial cells to VEGF-A at endothelial cell-endothelial cell boundaries (*contact-inhibited chemotaxis*) and increases the stability of those boundaries [18]. The growth rate of cultured endothelial cells decreases as the area of VE-cadherin junctions increases (*contact inhibition of growth*)[19].

Normal new blood vessels and tumor-induced blood vessels differ greatly in morphology and function. Normal new vessels recruit pericytes and vascular smooth-muscle cells to sites adjacent to the endothelial cells to stabilize the vessel [20]. Tumor-induced blood vessels often lack a hierarchical arrangement and have irregular diameters, high tortuosity, random branching, and defective endothelial barrier function [20]–[22].

### Computational Background

Because of the range of scales involved in cancer biology, cancer simulations employ a wide range of techniques depending on their biological focus. Standard partial-differential-equation (*PDE*) continuum models include scales down to the grid size used to solve the equations. Continuum multi-grid and adaptive-mesh techniques can cover scales from 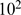 µm to 10 cm. Hybrid models, which use cellular automata, agent-based or multi-cell techniques to represent individual cells and continuum PDEs to represent diffusion of molecules, can capture more detail than continuum models spanning scales from microns to millimeters (for comprehensive reviews of mathematical models of tumor-growth and angiogenesis see [23]–[27] and references therein).

Zheng et al. [28] have used an adaptive finite-element/level-set method to model solid tumor growth in combination with Anderson and Chaplain's hybrid model of angiogenesis [24]. Zheng's model treats tumor cells as a viscous fluid flowing through a porous medium obeying the Darcy-Stokes law. Zheng et al. have shown that both diffusional instability (competition of growth and surface tension) and co-option of the new anastomosed capillaries may be key glioma invasion mechanisms. Frieboes et al. [29] have used Zheng's level-set method in combination with Plank and Sleeman's hybrid continuum-discrete [30], lattice-free model of tumor angiogenesis to model the physiology and evolution of glioma neovasculature in 3D. Frieboes et al's model allowed them to correlate measurable tumor microenvironment parameters to cell phenotypes and potentially to tumor-scale growth and invasion. Cristini et al. [31] have also developed a continuum model of solid avascular tumors using a mixture model obeying Cahn-Hilliard-type equations. Cristini et al. found that taxis of tumor cells up gradients of nutrient produces fingering instabilities, especially when tumor cell proliferation is slow.

The effects of blood flow on vascular remodeling and tumor growth have been extensively studied by Owen et al. [32], Alarcon et al. [33], Bartha and Rieger [34], Welter et al. [35], McDougall et al. [36], Stephanou et al. [37], [38], Pries et al. [39]–[42] and Macklin et al. [26]. Other vasculature and transport-related effects remain to be studied [43], for example how tumor cells interact with endothelial cells and enter and exit the blood stream (*intra/extravasation*) and spread to other organs (*metastasis*) via blood vessels. The simple model we present in this initial paper neglects the crucial effects of detailed transport due to blood flow and the effects of flow-induced vascular remodeling. We discuss some of the resulting artifacts and missing behaviors below.

Because the Glazier-Graner-Hogeweg model (*GGH*, also known as the Cellular Potts Model, *CPM*) [44]–[46] handles cell-cell and cell-ECM adhesion and cell motility more naturally than many other modeling methods, GGH simulations may provide additional insight into tumor growth and the complex roles of angiogenesis. Poplawski et al. [47] developed a GGH simulation of avascular tumor based on Anderson's model [48] to study the effects of adhesion and nutrient transport on the morphology of avascular tumors. Poplawski et al. found that nutrient-deprived tumors are generally more invasive and that high tumor-ECM surface tension changes seaweed-like tumor-morphologies into dendritic morphologies. Rubenstein and Kaufman [49] have developed a GGH model of avascular tumors with explicit representation of two types of ECM fibers to study the effect of ECM on growth of glioma spheroids *in vitro*. Rubenstein and Kaufman showed that invasion is maximized at intermediate collagen concentrations, as occurs experimentally.

In our simulations, the vascular structure produces inhomogeneities in oxygen tension on scales larger than those appearing in continuum simulations depending on inhomogeneities in Extracellular Matrix (*ECM*) and smaller than those due to tissue structure. These inhomogeneities may affect tumor growth rates and morphology and the somatic evolution of metastatic potential within tumors. Thus, simulating vascular structure at the cell level is crucial to developing biomedically-meaningful tumor simulations.

In this paper we simulate 3D solid tumor growth and angiogenesis using the multi-cell GGH model. Our simulation omits many biological details, but provides a useful starting point for the construction of more realistic models. We focus on tumors where the vasculature remains peripheral to the growing tumor. Our major simplifications include: 1) We neglect the distinction between veins and arteries, anastomosis, and the possible presence of pericytes and smooth muscle cells. 2) Since oxygen-depleted areas of a tissue coincide with nutrient-depleted and energy-depleted areas [50], we assume that oxygen serves as the single limiting substrate field. 3) Cells become hypoxic or necrotic by simple thresholding depending on the local concentration of oxygen. 4) We neglect the basal metabolic consumption of oxygen by tumor cells. 5) We assume that the oxygen concentration in the capillaries is constant along the blood vessels, neglecting vessel diameter, blood flow rate and vessel collapse due to external pressure. 6) We assume that oxygen diffuses uniformly in the host tissue and tumor. 7) We caricature the results of the hypoxic signaling pathways as constant-rate secretion by hypoxic cells of a single long-diffusion-length isoform of VEGF-A like VEGF-A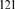. 8) Since we do not model blood flow explicitly, we neglect its biomedically important effects on vascular remodeling and the maturation of nascent blood vessels. 9) Rather than model tip-cell selection explicitly, we distribute a certain number of **inactive neovascular** cells (terms in bold, *e.g.* **neovascular,** refer to specific simulation classes), which behave identically to **vascular** cells until the concentration of VEGF-A exceeds an activation threshold. 10) We adopt the vascular-patterning hypothesis that activated vascular endothelial cells secrete and chemotax towards a short-diffusion-length chemoattractant (for a discussion of possible chemoattractant candidates see [51], [52]). 11) We employ a simplified model of tight junctions between endothelial cells in the preexisting vasculature. 12) We do not represent the ECM and the cells in the surrounding normal tissue explicitly and neglect mechanisms related to ECM and normal tissue remodeling, like secretion of ECM proteins by tumor cells, secretion of ECM-degrading enzymes (*matrix metalloproteinases*), and lactic acid accumulation.

## Results

**Figure 1:**
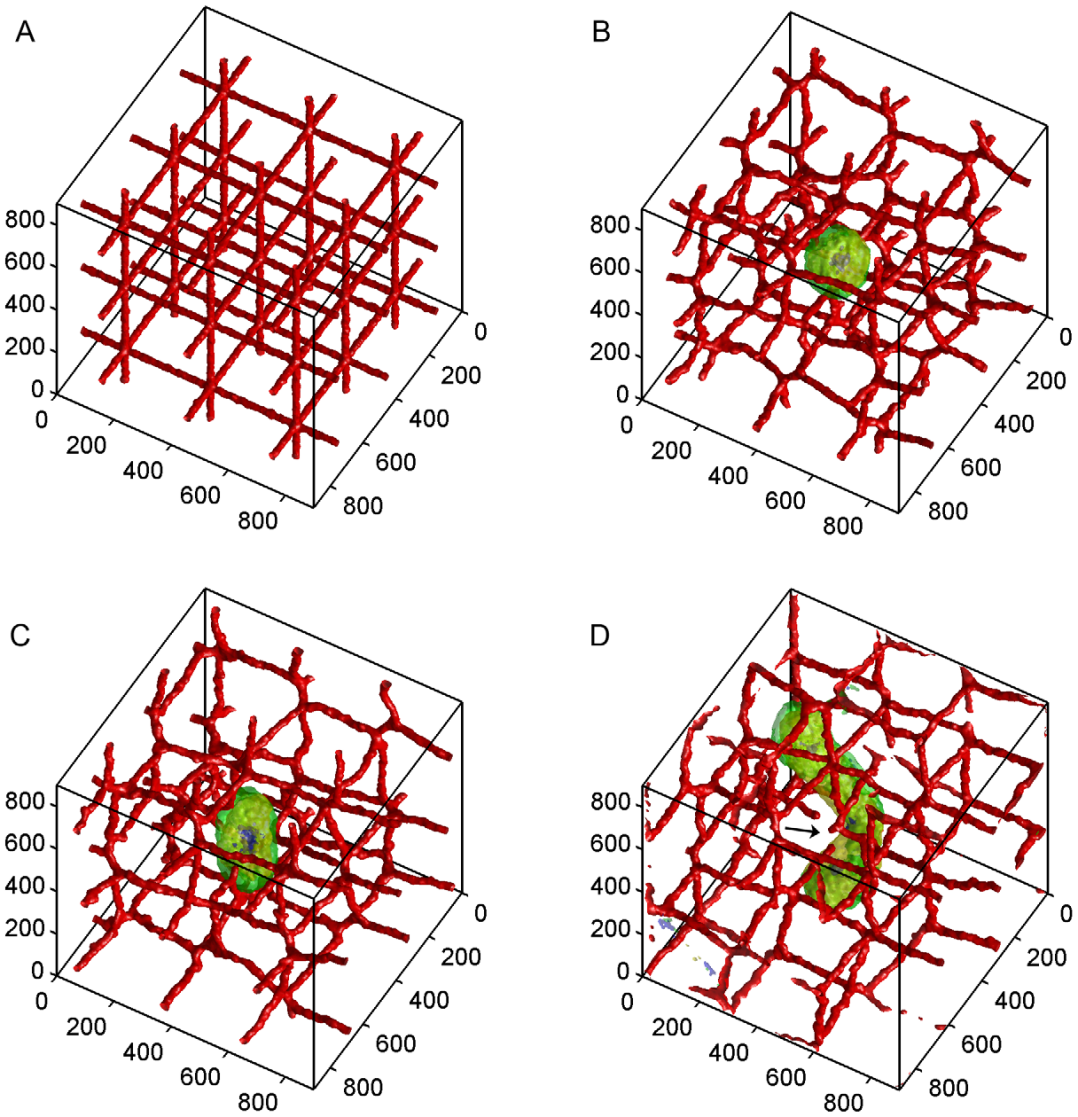
Time-series of simulated tumor growth without angiogenesis. A) Day 0: Pre-existing vasculature and the initial normal tumor cell at: *x* = 425 µm, *y* = 425 µm, *z* = 425 µm. B) Day 15: The tumor grows into a sphere with a maximum diameter of about 200 µm and remains at this size from day 10 to day 25. C) Day 30: The tumor grows into a cylinder with a diameter of about 200 µm and a length of about 300 µm. The vasculature is about to rupture. D) Day 75: The black arrow shows the location of the ruptured vessels. Cell types: Green: normal; yellow: hypoxic; blue: necrotic; red: vascular; purple: neovascular. Axes are labeled in µm.

**Figure 2:**
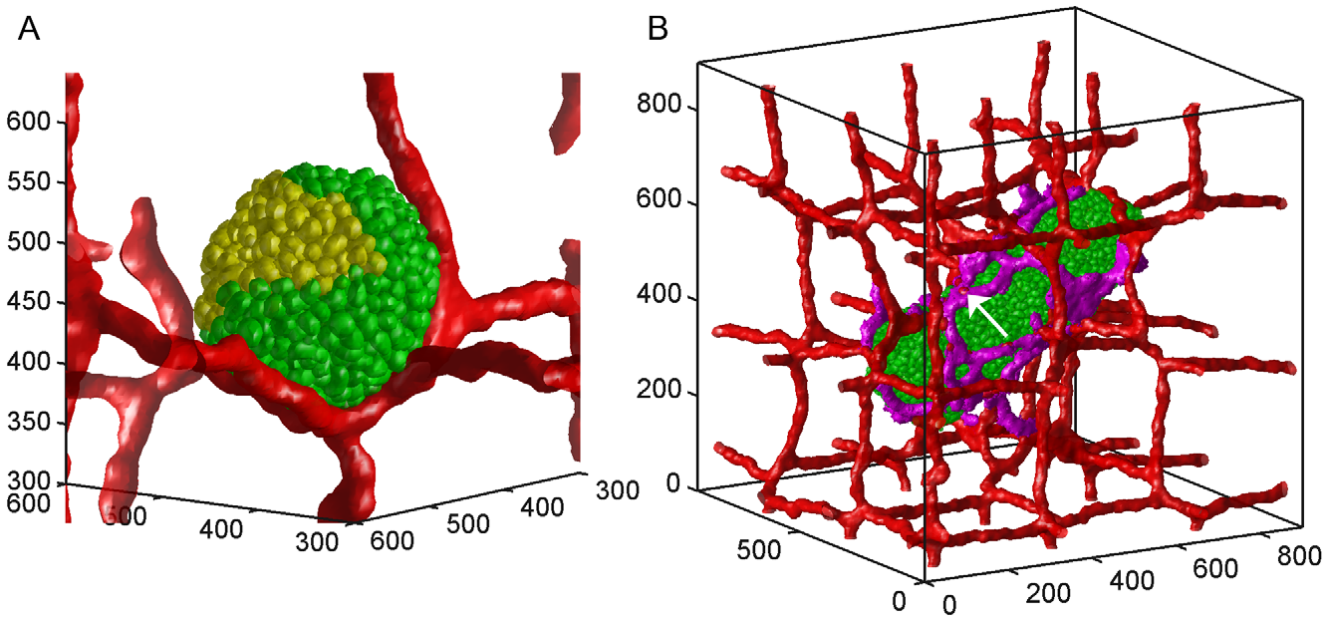
Single-cell rendering of tumor cells. The green cells are normal tumor cells and the yellow cells are hypoxic cells. The preexisting vasculature is rendered in red. A) Day 10: a spherical tumor without angiogenesis withnormal tumor cells only present near blood vessels. B) Day 60: A cylindrical tumor with angiogenesis, diameter 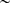
300 µm and length 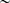
800 µm. The purple cells are active neovascular cells which are not rendered individually. The white arrow indicates a vascular cell incorporated into a neovascular branch. Axes are labeled in µm.

**Figure 3:**
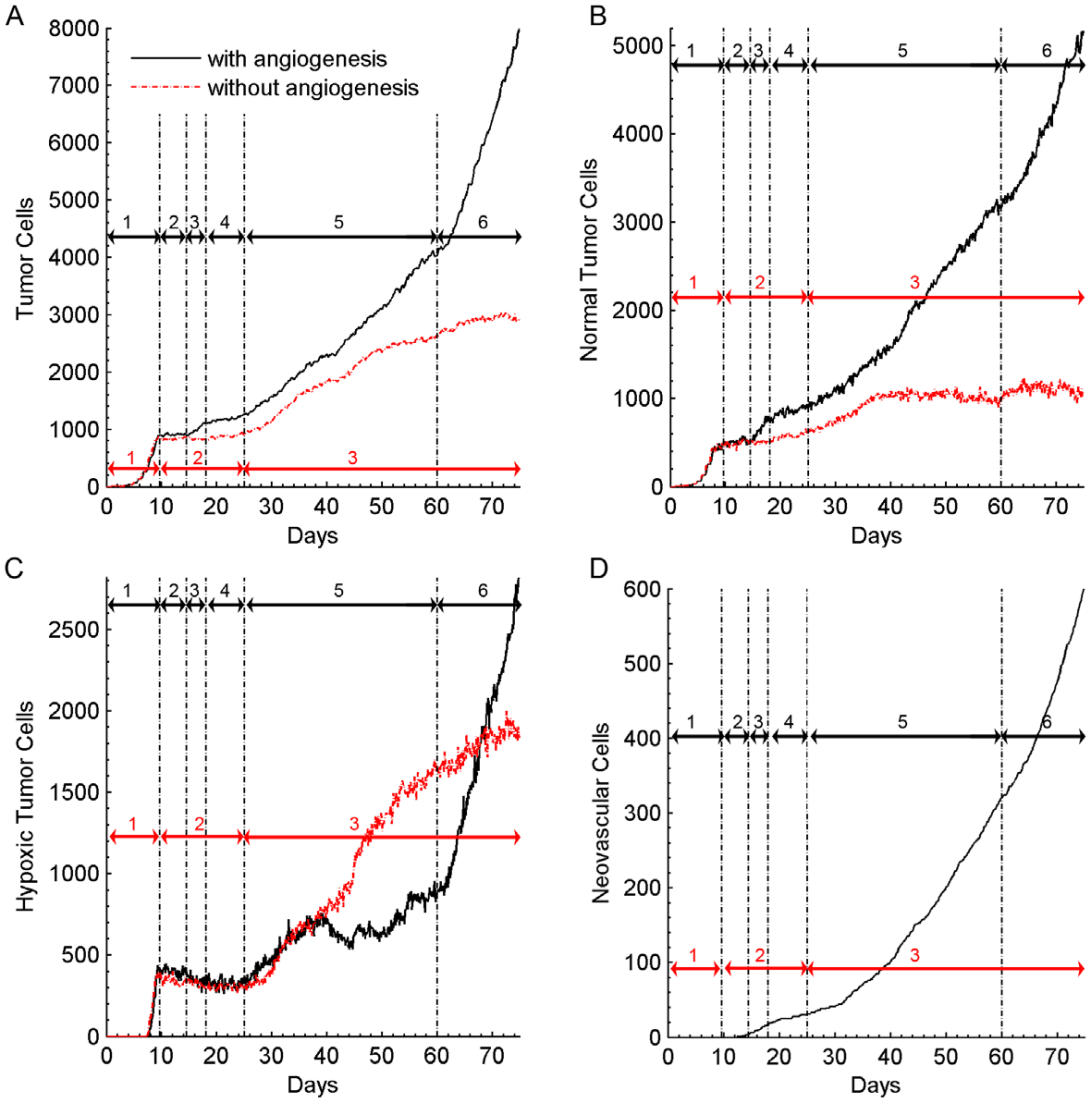
Growth curves for simulated tumors with (black) and without (red) angiogenesis. Black arrows: (1) the exponential growth phase of the spherical tumor; (2) no growth; (3) the linear-spherical phase; (4) slow growth; (5) the linear-cylindrical phase; (6) the linear-sheet phase. Red Arrows: (1) the exponential growth phase of the spherical tumor; (2) slow growth; (3) cylindrical growth phase. A) The number of live tumor cells (normal and hypoxic) during 75 days of simulated tumor growth with and without angiogenesis. B) Development of the number of normal tumor cells *vs.* time. C) The number of hypoxic tumor cells *vs.* time. D) The number of neovascular cells in the simulation with angiogenesis *vs.* time.

We ran otherwise identical simulations with and without angiogenesis to study how tumor-induced vasculature affects tumor growth and morphology. Figure 1 shows a time-series of a growing tumor without angiogenesis. The tumor grows exponentially for the first 10 days (Figure 2A and Figure 3A, region 1 red); growth then slows and the tumor reaches a diameter of about 200 µm (Figure 1B). **Hypoxic** domains form on day 8 (Figure 3C) and **necrosis** begins 12 hours later. The tumor grows slowly and remains almost spherical until day 25 (Figure 3A, region 2 red). At this stage, **normal** tumor cells form a layer of maximum thickness 75 µm near blood vessels and no **normal** cells persists far from the vasculature. The oxygen inhomogeneity produces tumor protrusions towards the adjacent blood vessels. Eventually the growing tumor pushes and stretches the vessels it contacts, finally rupturing them on day 31 (see Movie S1 and Figure 1C). At this stage, the tumor is large enough to span the gaps between vessels and thus to access new vessels. This strategy allows the tumor to grow at a moderate (Figure 3A, region 3 red) rate throughout the 75 day simulation even without angiogenesis and produce a cylindrical tumor with a diameter of about 200 µm following the path of nearby blood vessels with **normal** cells only present near blood vessels. This avascular tumor growth is reminiscent of the first and second stages of growth of gliomas [53] although glioma cells usually form a peripheral cluster encompassing the blood vessel. (The growth and morphology of the tumor after day 31 is an artifact of the simplified oxygen transport in our model, see Discussion).

**Figure 4:**
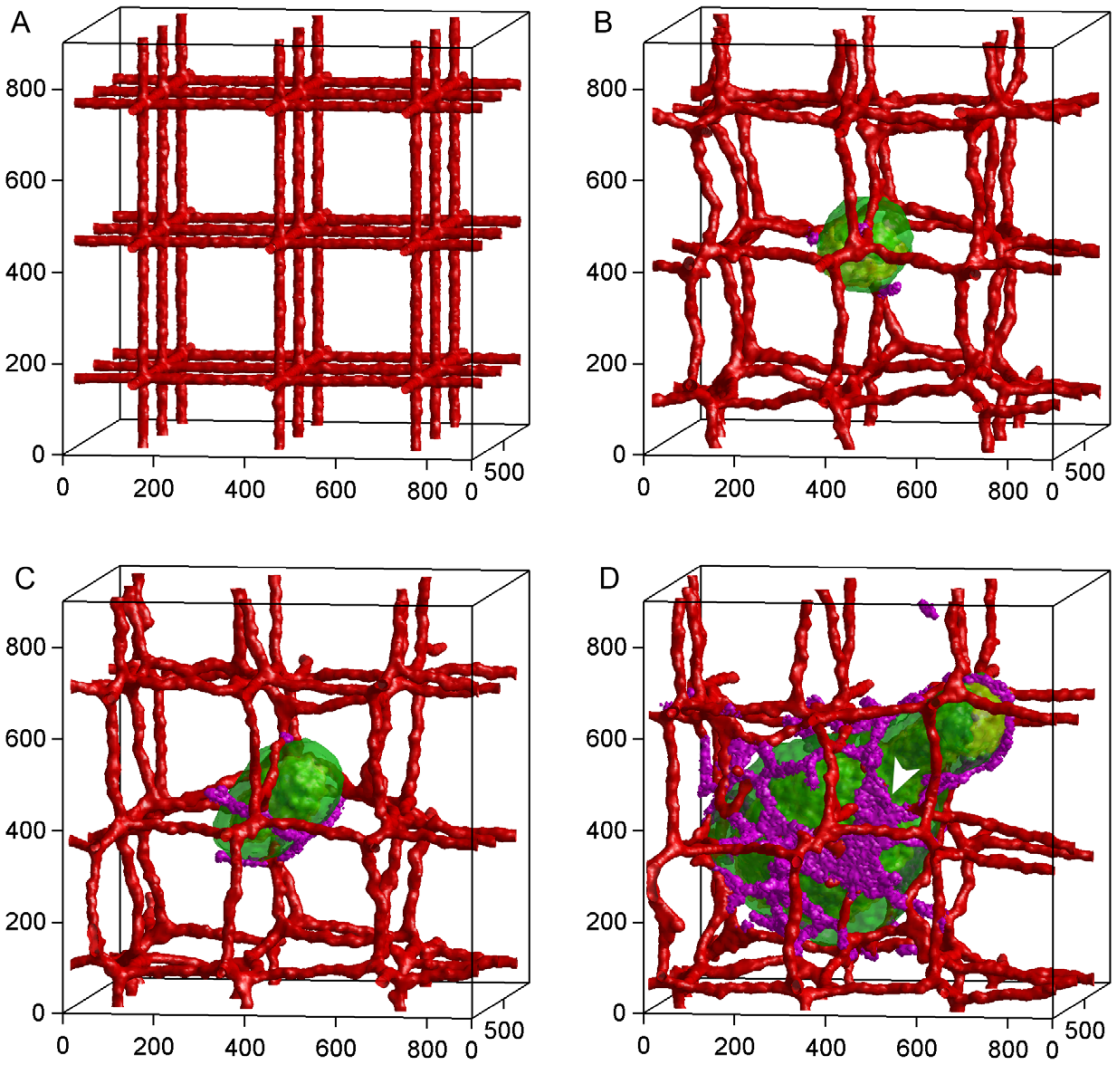
Time-series of tumor growth with angiogenesis. A) Day 0: The pre-existing vasculature and the initial normal tumor cell. B) Day 15: The tumor grows into a sphere with a maximum diameter of about 300 µm. The purple cells are active neovascular cells. C) Day 30: The tumor grows into a cylinder with a length of about 350 µm and a diameter of about 300 µm. The vasculature is about to rupture. D) Day 75: The developed vascularized tumor. The white arrow-head shows neovascular cells organized into 2D sheets. Cell types: Green: normal; yellow: hypoxic; blue: necrotic; red: vascular; purple: neovascular. Axes are labeled in µm.

In simulations with angiogenesis, tumor growth resembles that without angiogenesis for the first 15 days. Figure 4 shows the time evolution of the number of living tumor cells in representative examples of both types of simulations. Growth is initially exponential (3A, region 1 black). On day 8, **hypoxic** domains form and start secreting VEGF-A (Figure 3C). VEGF-A reaches and activates the nearest inactive **neovascular** cells a few hours later. Activated **neovascular** cells then proliferate and chemotax up the VEGF-A gradient. The elastic attachment of the initial **neovascular** cells and contact inhibition of growth slow the cell-cycle time of **active neovascular** cells in the preexisting vasculature to 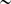
4 days (Figure 3C). Daughter **neovascular** cells which lack elastic links and can have less contact with neighbors have cell cycles of 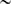
1–2 days. The tumor does not grow significantly from day 10 to day 14 when the first angiogenic sprouts appear (Figure 3A, region 2 black). **Neovascular** cells form a connected peri-tumor network about 12 days after activation. Figure 3A shows that the induced peri-tumor vasculature results in a phase of linear tumor growth which we call the *linear-spherical phase* (Figure 3A, region 3 black). During this phase, the tumor grows linearly in volume, remaining spherical until it reaches a diameter of about 300 µm on day 18. Growth then slows drastically from day 18 to 25 (Figure 3A, region 4 black). After day 25 the larger tumor is more sensitive to inhomogeneities in cell proliferation, initiating a second phase of linear growth as tumor changes from spherical to cylindrical (the *linear-cylindrical phase*, Figure 3A, region 5 black). This change in shape can be interpreted as the growth of the mode with the longest unstable wavelength which allows the tumor to grow indefinitely as long as the peri-tumor vasculature covers the entire tumor. By day 60, the cylindrical tumor has a bigger diameter than that in the simulation without angiogenesis, with **normal** cells distributed symmetrically about its axis. Positive feedback between **hypoxic** cells and **neovascular** cells results in extensive growth of **neovascular** cells. **Neovascular** cells self-organized into 2D vascular sheets instead of 1D vascular branches at **neovascular** densities higher than a critical density. These thicker branches initiate a third phase of *linear-sheet* growth on day 60 (Figure 3A, region 6) as the cylindrical tumor (Figure 2B) grows into a paddle-like structure (Figure 4D).

## Discussion

In general, tumors in our simulation are smaller than those studied by Macklin et al. [26]. Macklin et al. found that avascular tumors remained spherical during 45 days of simulated growth, independent of the possible production or degradation of ECM. In our simulations, the avascular tumors become more cylindrical after day 25 and grow along (parallel to) the nearest blood vessel. The models are sufficiently different that we cannot yet identify the cause of this discrepancy. Both models produce similar growth during the first 25 days and the hypoxic core forms about the same time in both models (compare Figure 3A–C, region 2 and 3 red to Figure 3 and 11 from [26]). In both our simualtions with angiogenesis and Macklin et al. 's (vascular growth simulation with the effect of solid pressure-induced neovascular responce and enhanced ECM degredation from [26]) the new vasculature remains outside the tumor, which becomes elongated and paddle-shaped. Vascular glioma tumors in Zheng et al. 's model [28], like avascular tumors in our model, show co-option behavior although gliomas in their model breaks up into fragments and encompasses newly formed blood vessels.

Our simulations assume that angiogenic sprouts can partially support oxygen transport even before anastomosis. This assumption affects tumor growth between days 15 and 20 when most vascular sprouts have not formed closed loops. Requiring anastomosis for oxygen transport would both delay the onset of the vascular phase of tumor growth and make that growth more rapid once it starts. Later in the simulation, most neovascular cells form closed loops, so our simplification should have less effect. Another artifact of our model of oxygen transport is that the tumor in the simulation without angiogenesis grows to a size comparable to the one in the simulation with angiogenesis, albeit more slowly. In the simulation without angiogenesis, inhomogeneity in tumor-cell proliferation exerts a force on the preexisting vasculature which can stretch and even rupture vascular cords (Figure 1D, Movie S1). Since we do not consider blood flow, such vascular damage does not change 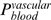 (oxygen transport). If we calculated blood flow, the tumor would stop growing on day 31, then shrink slightly due to lack of oxygen. Vascular ruptures also happen in the simulation with angiogenesis (Figure 2B), but **neovascular** cells usually form new vessels to fill the gaps.

In reality, oxygen supply depends on blood vessel characteristics like diameter and length. Because the production of O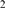 in our simulations is proportional to the number of voxels in blood vessels, more **ECs** supply more oxygen, regardless of their organization. For example, formation of thick vascular cords and 2D vascular sheets in the simulation with angiogenesis (Figure 1D, Movie S1) on day 60 initiates a phase of fast linear growth which is an artifact of our simplified oxygen transport. Including the effects of vascular-network connectivity and depletion of oxygen along the direction of blood flow would produce more realistic tumor growth and morphology.

In our simulations, the preexisting vasculature with an average vascular branch length of about 300 µm supplies oxygen initially. Vascular networks with shorter average vascular branch lengths (keeping the average partial pressure of oxygen the same) would produce larger solid tumors both with and without angiogenesis. Since a tumor can grow to a maximum diameter of 200 µm without angiogenesis, average vascular branch lengths shorter than 200 µm will produce infinitely long cylindrical solid tumors.

The random motility of **normal** and **hypoxic** cells within the tumor also affects growth, reducing the inhomogeneities in cell proliferation which change spherical tumors into cylinders. Higher cell motilities paradoxically arrest tumor growth at diameters of 200 µm (no angiogenesis) and 300 µm (with angiogenesis) (data not shown). In contrast, lower cell motilities enhance formation of fingers and nodules which increase the invasiveness of the solid tumor. In reality, motility also depends on the adhesiveness of cells to each other and to the ECM, so this effect may not be clinically relevant.

We also assume that the vasculature remains peripheral to the tumor. Formation of blood vessels inside the tumor enhances oxygen transport and allows arbitrarily large tumors and faster, though not necessarily more invasive, growth.

Tumor growth in real tissues leads to increasing hydrostatic and solid pressures, inducing tumor-cell quiescence and necrosis and also causing blood vessels to collapse. Because **ECM** in our simulation is not confined by physical boundaries and does not have a volume constraint, the pressure in our tumor does not change due to tumor growth. Explicitly modeling the cells and ECM in the peri-tumor region would result in a more realistic pressure.

Our simplified tumor induces angiogenesis through VEGF signaling, but neglects other tumor-induced changes in the surrounding vasculature, including apoptosis of endothelial cells because of reduced pericyte coverage and lowered pH. Combining these vascular remodeling mechanisms with blood-flow calculations would give further insight into the effects of angiogenesis on vascular tumor growth.

Although our high-resolution simulation represents a fairly small tissue volume, our results can scale to treat larger tissue volumes at lower spatial resolution. Such scaling tradeoffs may be useful for simulating tumors like prostate cancer, which involve multiyear progressions and centimeter-scale tissues.

A real solid tumor with a volume of about 1 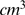 typically has 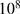 cells. Our current version of CompuCell3D (see Implementation Parameters and Initial Conditions) can simulate 75 days of growth of a tumor containing 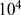 cells in 7 days on a single-core processor. To simulate more realistic tumors without needing to treat cluster of cells as single generalized cells, we are investigating parallel computation techniques. Chen et al. [54] have developed a Message Passing Interface Standard (*MPI*) parallel implementation of the GGH which permits simulations with more than 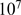 cells. Currently, we are developing a parallel implementation of CompuCell3D to take advantage of multi-core processors with shared memory and graphics processing units. The new version of CompuCell3D will be able to simulate more than 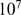 cells in 7 days.

Our next extension of these simulations will include blood flow to improve our description of substrate transport and also include shear effects on vascular remodeling.

## Methods

### The Glazier-Graner-Hogeweg Model

Our simulation uses the Glazier-Graner-Hogeweg model (*GGH*, also known as the Cellular Potts Model, *CPM*), a multi-cell, lattice-based, stochastic model which describes biological cells and their interactions in terms of *Effective Energies* and constraints. GGH applications include modeling avascular tumor growth [47], [49], biofilms [55], chick limb growth [56], somitogenesis [57], blood flow and thrombus development [58] and angiogenesis [51], [59]–[61]. Each cell consists of a domain in a *cell lattice* of *lattice sites*, or *voxels*, at locations 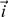, sharing the same *cell index*, 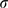. Each cell has an associated *cell type*, 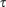. The *Effective Energy* governs changes in the cell configuration. The basic *Effective Energy* describes constant-volume cells interacting via differential adhesion:
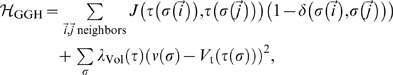
(1)where 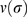 is the total number of lattice sites in cell 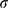, which is constrained to be close to the target volume 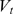 (*i.e.*, deviation of 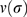 from 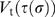 increases the *Effective Energy*), and 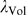 is the inverse compressibility of cells of type 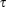. 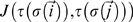 is the contact energy per unit area between cells 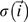 and 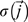. 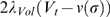 is the internal pressure in cell 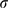. To simulate the cytoskeletally-driven reorganization of cells and tissues, we model cell protrusions and retractions using a *modified Metropolis dynamics*. For each attempt of a cell to *displace* a neighbor, we select at random a cell boundary (*source voxel*) and a neighboring *target voxel* and calculate the change in the effective energy 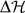, if the source cell displaced the target cell at that voxel. If 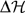 is negative, *i.e.*, the change is energetically favorable, we make it. If 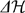 is positive, we accept the change with probability 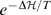, where 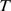 describes the amplitude of cytoskeletal fluctuations. On a lattice with 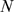 sites, 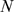 displacement attempts represent our basic unit of time, one *Monte Carlo Step* (*MCS*). We describe secreted morphogens and oxygen macroscopically by concentration **fields** discretized at the resolution of the cell lattice. **Fields** evolve according to simple time-dependent partial-differential equations (*PDEs*). In addition to contact and volume energies, we include a chemotaxis term in the Effective Energy to model angiogenesis (see below).

We define a special, generalized cell representing the extracellular medium **(ECM)**. The total volume and surface area of **ECM** are not constrained. **ECM** voxels can be both source voxels, *e.g.* during retraction of the trailing-edge of a cell, and target voxels, *e.g.* during formation of lamellipodia. Since ECM does not have cytoskeletal fluctuations, we use the amplitude of cytoskeletal fluctuations of the target or source cell to determine the acceptance of the displacement.

Our model contains three tumor-cell types: **normal,** **hypoxic** and **necrotic** (we use the designation **tumor** cell to refer to both **normal** and **hypoxic** cells). **Normal** tumor cells become **hypoxic** when the oxygen partial pressure (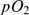) is less than 5 mmHg [22], [50], [62] and become **necrotic** when 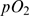 drops below 1 mmHg. **Normal** and **hypoxic** cells take up oxygen (see below) and proliferate at a rate which depends on the oxygen partial pressure according to a Michaelis-Menten form. To model **tumor cell** proliferation, we increase the cell's target volume according to:
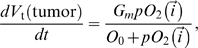
(2)where 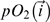 is the 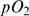 at the center-of-mass of the cell. We discuss the *maximum growth rate*, 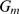, in the next section. When a cell's volume reaches its *doubling volume*, we split the cell along the *x–y* plane. The two daughter cells receive equal target volumes of half of the target volume of the mother cell at mitosis. **Necrotic** cells lose volume at a constant rate:
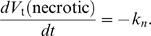
(3)


We remove zero-volume cells. **Hypoxic** cells secrete the **field** 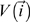, which models the pro-angiogenic factor VEGF-A. **Hypoxic** cells stop secreting VEGF-A and become **normal** if 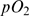 is above 5 mmHg and become **necrotic** if 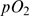 drops below 1 mmHg. **Hypoxic** cells secrete 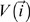 at a constant normalized rate 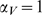 per MCS at each voxel in their volume. 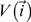 diffuses with diffusion constant 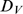 and decays at a rate 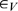, so 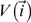 evolves according to:
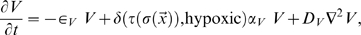
(4)where 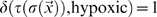 at voxels belong to **hypoxic** cells and 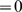 elsewhere. Since cell motility is large, cells diffuse and rearrange fast enough to prevent artifacts due to their fixed cleavage plane [47].

Our model also contains two basic **endothelial** cell **(EC)** types: **vascular** and **neovascular.** We further distinguish two types of **neovascular** cells, **inactive neovascular** and **active neovascular.** **Vascular** cells build the preexisting mature vasculature. To represent tight junctions between endothelial cells in mature capillaries which maintain the integrity of blood vessels, linear springs connect the centers-of-mass of **vascular** cells. The springs obey the elastic constraint, 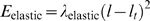, where 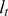 is the equilibrium length of the connection and 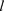 is the distance between the two neighbors. To model vascular rupture we set 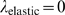 between the two neighbors when 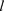 between the two neighbors is greater than 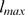. **Inactive neovascular** cells behave exactly like **vascular** cells. However, above a threshold value 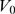 of 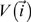 **inactive neovascular** cells switch irreversibly into **active neovascular** cells. **Active neovascular** cells can proliferate and chemotax up gradients of VEGF-A. To account for the contact-inhibited growth of **neovascular** cells, when the common surface area with other **vascular**, **inactive neovascular** and **active neovascular** cells is less than a threshold, we increase the target volume of the **active neovascular** cells according to the Michaelis-Menten relation:
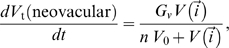
(5)where 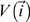 is the concentration of VEGF-A at the center-of-mass of the cell and 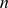 is a scaling constant describing the proportionality of the activation concentration to the concentration at which the growth rate is half its maximum. **Active neovascular** cells divide along the *x–y* plane when their volume reaches a specified *doubling volume*. After mitosis, both daughter cells are **active neovascular** and inherit half of their mother's target volume. **Active neovascular** cells at the tip of a sprout share two features with real tip-cells: 1) Compared to stalk cells, they have more free boundary which can respond to gradients of VEGF-A, dragging the rest of the sprout up the gradients. 2) Dragging reduces the contact area between **active neovascular** cells, promoting neovascular growth. Unlike real tip-cells, **active neovascular** cells at the tip of a sprout proliferate. Thus sprouts grow faster in steeper gradients of VEGF-A as long as the concentration of VEGF-A is well below the saturation concentration. We add a saturated Savil-Hogeweg-type chemotaxis term with contact inhibition to the basic 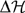 of the Effective Energy to represent the net effect of preferential formation of pseudopods in response to the gradient of the chemoattractant **field** near the **active neovascular** cell's boundary [63]:
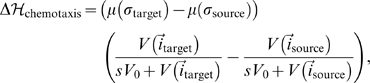
(6)

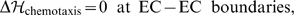
(7)

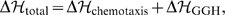
(8)where 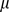 is the degree of chemotactic response of the cell and *s* is a positive number which scales the VEGF-A concentration **field** relative to the **neovascular** activation threshold. 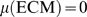.

Growth and chemotaxis of **active neovascular** cells up gradients of VEGF-A produce a dispersed growing population of **neovascular** cells rather than a connected capillary network [51]. To self-organize **vascular** and **neovascular** cells into a capillary-like structure we extended to 3D Merks' 2D model of angiogenesis in which endothelial cells self-organize into capillary-like networks in response to *autocrine* chemotaxis to a very short-diffusing chemoattractant [51]. We denote this chemoattractant **field** by 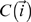. **Vascular** and **neovascular** cells secrete it at a constant rate 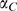 at all their voxels, the chemoattractant degrades at a constant rate 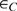 in the **ECM,** and diffuses at a constant rate 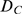 everywhere:
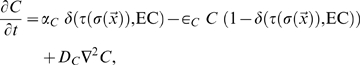
(9)where 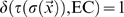 inside **vascular** and **neovascular** cells and 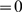 elsewhere. Both **vascular** and **neovascular** cells in our model chemotax up gradients of 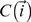. We include a linear version of eq. 8 [51] to model contact-inhibited chemotaxis:
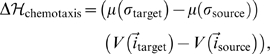
(10)

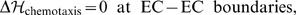
(11)

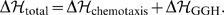
(12)


Autocrine chemotaxis produces a capillary-like structure, while the elastic constraint between **vascular** cells makes the preexisting vasculature more stable and less flexible.

Vasculature transports oxygen to the host tissue and the tumor. We represent the oxygen partial pressure, 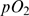, by a **field** 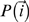. Since we initialize our simulations with a fully anastomosed preexisting vasculature and since not all **neovascular** cells form in closed loops, we assume that the oxygen partial pressure in the preexisting capillaries, 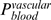, is higher than 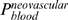 in the tumor-induced vasculature (see [64], [65], for more accurate blood-flow calculations). We assume that the oxygen **field** concentration neighboring a vessel changes proportionally to 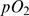 according to a solubility coefficient, 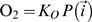. Available 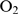 diffuses at a constant rate 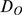 in the surrounding tissue and is consumed by **tumor cells.** Since oxygen consumption of human cells is almost constant until 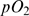 drops below 0.5–1 mmHg and the consumption rate is constant below this pressure, we use a piecewise-linear approximation of Michaelis-Menten kinetics to model oxygen consumption. **Tumor** cells take up oxygen at a rate proportional to 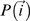 with a maximum rate of 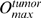. The cells near the vasculature consume oxygen at their maximum rate, but cells far from the vasculature have growth limited by the available oxygen and take up oxygen at a rate 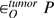. To represent oxygen consumption by host cells in the normal tissue, which we do not model explicitly, we assume an oxygen consumption rate of 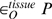 saturating at a maximum rate of 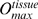 in the **ECM.** We include oxygen consumption by **EC** cells in the adjusted oxygen partial pressure. Thus oxygen evolves according to:
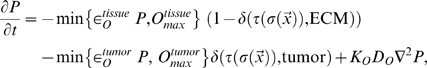
(13)

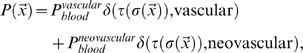
(14)where 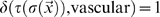 and 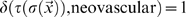 inside **vascular** and **neovascular** cells and 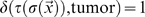 while 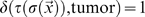 and 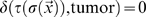 elsewhere. 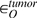 is the oxygen consumption rate for both **normal** and **hypoxic** cells. We have summarized the properties of the **fields** in Table 1 and cell types and their behaviors in Table 2.

**Table 1:** Diffusive molecules in the vascular tumor-growth simulation.

Fields	Definition	Role/Properties
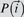	partial pressure of oxygen	-regulates tumor cell growth
		-induces normal 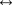 hypoxic transition
		-induces hypoxic 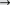 necrotic transition
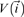	long-diffusing proangiogenic factor	-hypoxic signaling
		-induces inactive neovascular 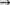 active neovascular transition
		-regulates neovascular growth
		-chemoattractant for vascular and neovascular cells
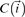	short-diffusing chemoattractant	-self-organizes vascular and neovascular cells into capillary networks
		-chemoattractant for vascular and neovascular cells

All molecules diffuse everywhere uniformly and isotropically. Boundary conditions of the lattice are periodic.

**Table 2:** Cell types in the simulations and their behaviors.

Cells	Behaviors
Tumor cells	
Normal	-proliferate
	-consume oxygen
	-change to hypoxic
	-change to necrotic
Hypoxic	-proliferate
	-consume oxygen field
	-change to normal
	-change to necrotic
	-secrete long-diffusing proangiogenic field 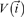
Necrotic	-shrink
	-disappear
Endothelial cells	
Vascular	-consume oxygen field
	-supply oxygen field at partial pressure 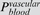
	-secrete short-diffusing chemoattractant field 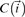
	-chemotax up gradients of field 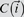
	-elastically connect to neighboring
	vascular and inactive neovascular cells
	-lose elastic connections, when 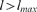
Inactive neovascular	-consume oxygen field
	-supply oxygen field at partial pressure 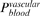
	-secrete short-diffusing chemoattractant field 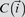
	-chemotax up gradients of field 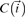
	-elastically connect to neighboring
	vascular and inactive neovascular cells
	-lose elastic connections, when 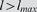
	-change to active neovascular
Active neovascular	-consume oxygen field
	-supply oxygen field at partial pressure 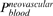
	-secrete short-diffusing chemoattractant field 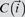
	-chemotax up gradients of field 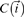
	-chemotax up gradients of field 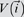
	-proliferate

### Implementation Parameters and Initial Conditions

Our simulations use the open-source CompuCell3D simulation environment (http://www.compucell3d.org/) [66]. We ran our simulation on a 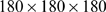 lattice with periodic boundary conditions. One voxel is equivalent to 125 µm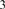. The cell lattice represents a tissue with a volume of 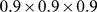 mm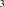. Our average simulated **tumor** cell has a volume of about 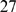 voxels or 3375 µm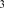. We stored the cell-lattice configuration every 3 simulated hours and rendered each snapshot using the MATLAB volume-visualization functions (Our group is integrating post-rendering capabilities into CompuCell3D). Since rendering individual cells is computationally expensive, in Figures 1 and 4 and Supplemental Movies (Movie S1 and S2) we have only rendered boundaries between cells which differ in type. For demonstration purposes, we have rendered the boundaries of individual **tumor** cells in the simulation with angiogenesis on day 60 (Figure 2B) and in the simulation without angiogenesis on day 10 (Figure 2A).

Experimentally, tumor cells from lines such as U-87 human glioma [67]–[70] can move at a rate of about 0.35 µm/min. For typical parameter settings in our simulations **tumor cells** move at about 0.1 pixels/MCS. Equating the experimental and simulated mean cell speeds implies 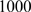 MCS 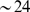 h.

We start our simulation with a single **normal** tumor cell near the center of the cell lattice and a preexisting network of blood vessels (see Figure 1). We assume that 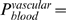
90 mmHg in the preexisting vasculature and 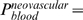
50 mmHg in the tumor-induced vasculature. We set the diffusion constant of oxygen in our simulations to 10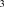 µm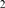/s, about half its diffusion constant in water. Since **hypoxic** cells in our simulations become **necrotic** if 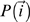 drops below 1 mmHg, we set both 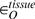 and 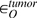 to 1. We set 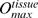 so the average 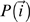 of the tissue reaches an asymptotic value of 20 mmHg. We assume that the density of cells in the tumor is about 10 times the density of the cells distributed in the **ECM** (which we do not represent explicitly). We assume that both **hypoxic** and **normal** tumor cells in our model consume oxygen at a rate up to 3 times that of the cells in the surrounding tissue, thus 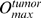 = 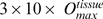. Higher oxygen consumption results in shorter oxygen penetration lengths and smaller solid tumors. We set 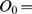
10 mmHg and choose 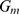 so that the cell cycle of **tumor** cells at 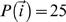 is 24 h. For these parameters our simulations produce solid tumors with maximum diameters of 200 µm.

Experimentally, the VEGF-A diffusion constant is about 10 µm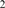/s in typical tissues and its decay rate is about 0.65 h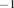 [52]. We set the activation VEGF-A concentration 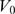 = 0.5, 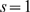, and pick 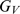 so that a **neovascular** cell that does not contact any **vascular** or **neovacular** cells has a minimum cell-cycle time of 24 h. Due to contact-inhibition of growth, **neovascular** cells incorporated in a tumor-induced vessel grow at a negligible rate.

We assume that integrins are down-regulated in **tumor** cells and that cell-cell adhesion via cadherins keeps the tumor solid, *i.e.* that the surface tension at the **tumor-ECM** interface is positive, 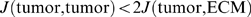 (for definitions of surface tensions see [44], [45]). We set a positive surface tension between **EC** **(vascular** and **neovascular)** and **tumor** cells (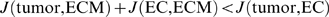) which keeps the vasculature peripheral to the tumor. For the specific values of *J* and the other parameters, see the supplemental *XML* and *python* files (Code S1).

## Supporting Information

Code S1 XML configuration and python scripts of the simulation with angiogenesis.
(0.27 MB ZIP)

Movie S1 Initially, the tumor grows exponentially and reaches a maximum diameter of about 200 micrometers. The growing cylindrical tumor pushes and stretches the vessels it contacts, finally rupturing them on day 31. Total movie time = 75 days. Time interval between frames = 6 hours. The camera rotates at a rate of 0.1 degree per frame. Cell types: Green: normal; yellow: hypoxic; blue: necrotic; red: vascular. Axes are labeled in micrometer.
(9.96 MB AVI)

Movie S2 Initially, the tumor grows exponentially. When oxygen level drops below a threshold, the normal tumor cells become hypoxic and start secreting a long-diffusing proangiogenic factor. At this stage, the tumor reaches a maximum diameter of about 300 micrometers. The activated neovascular cells in the pre-existing vasculature respond to the pro-angiogenic factors both by chemotaxing towards higher concentrations of the proangiogenic field and by forming new blood vessels via angiogenesis. The tumor-induced neovascular network enhances oxygen supply and tumor growth. Total movie time = 75 days. Time interval between frames = 6 hours. The camera rotates at a rate of 0.1 degree per frame. Cell types: Green: normal; yellow: hypoxic; blue: necrotic; red: vascular; purple: neovascular. Axes are labeled in micrometer.
(9.71 MB AVI)
